# Financial Risk Protection and Unmet Healthcare Need in Russia

**DOI:** 10.34172/ijhpm.2021.72

**Published:** 2021-07-14

**Authors:** Zlatko Nikoloski, Jane Cheatley, Elias Mossialos

**Affiliations:** Department of Health Policy, London School of Economics and Political Science, London, UK.

**Keywords:** Catastrophic Healthcare Expenditure, Out-of-Pocket Payments, Access to Healthcare, Russian Federation

## Abstract

**Background:** Achieving universal health coverage (UHC) includes financial risk protection. To date, catastrophic healthcare expenditure (CHE), the impoverishing effect of out-of-pocket (OOP) healthcare payments, and unmet healthcare need are the most widely used indicators for assessing the financial risk protection of a healthcare system. This study aimed to estimate the Russian healthcare system’s financial risk protection by focusing on CHE, OOP and unmet healthcare need.

**Methods:** The study used eight waves of the Russia Longitudinal Monitoring Survey (RLMS) (2010-2017) to analyze the financial risk protection of the Russian healthcare system. Commonly used indicators – CHE, both incidence and intensity, the impoverishing effect of CHE and unmet need –were used.

**Results:** We found low incidence and intensity of CHE in the Russian Federation. Our results are robust to various definitions of CHE (eg, as a share of total household expenditure or total household income). Furthermore, the impoverishing effect of OOP healthcare payments remains limited, despite the most recent economic slowdown (2014– 2016). This could be explained by a noticeable reduction in CHE during the crisis years, as postponing healthcare was adopted as a coping mechanism, particularly among households heavily affected by the crisis.

**Conclusion:** As stressed by the UHC framework, our findings suggest that CHE only partly captures inefficiencies and inequities in coverage, because one tenth of households forwent medical care for medicines and certain services. As spending on medicines and dental care are the main drivers of CHE, policy interventions should focus on extending coverage for pharmaceutical and dental care and target financial barriers to seeking care, particularly for the poor and vulnerable.

## Introduction

**Key Messages**

** Implications for policy makers**

- Based on eight waves of Russia Longitudinal Monitoring Survey (RLMS) (2010-2017), the incidence and intensity of catastrophic healthcare expenditure (CHE) in the Russian Federation are low.
- While poverty rates increased during the most recent slowdown, the impoverishing effect of out-of-pocket (OOP) has been limited.
- While CHE in Russia is low, a tenth of all households report unmet need for healthcare services.
- Unmet need for healthcare services is mostly concentrated in dental services and medications.

** Implications for the public**

We use eight waves of the Russian Longitudinal Monitoring Survey (2010-2017) to provide an in-depth analysis of the Russian healthcare system. We derive the following indicators: catastrophic healthcare expenditure (CHE), overshoot (and mean positive overshoot), impoverishing effect of OOP, and unmet need. The prevalence of CHE and the related metrics in the Russian Federation are low. Furthermore, the impoverishing effect of OOP healthcare payments remains limited, despite the effects of the most recent economic slowdown (2014-2016). These findings suggest that postponing accessing health services was adopted as a coping mechanism. We also observed that some households reported unmet need (particularly in the area of dental services and medications), suggesting that CHE only partly captures inefficiencies in healthcare coverage. Policy interventions should focus on extending coverage for dental and pharmaceutical care and target financial barriers to seeking care, particularly for the poor and vulnerable.

The macroeconomic recovery of the Russian Federation since the early 2000s, mainly driven by high global demand for and high prices of hydrocarbons, has had positive spillover effects on many social indicators. Since the Russian debt crisis in 1998, poverty rates have fallen, while average incomes have increased.^1^ The increase in incomes coupled with increased public health spending has also contributed to improvements in health outcomes. More specifically, the government expenditure on health (as a share of gross domestic product) has increased from 2.7% in 2004 to 3.2% in 2018.^1^ Life expectancy has improved, driven by a fall in mortality, particularly mortality due to cardiovascular and other non-communicable diseases.^1^ Health improvements could also be due to advances towards universal health coverage (UHC) and, in that respect, improving financial risk protection (measured by catastrophic healthcare expenditure – CHE) has been one of the main UHC targets.^2^ As CHE in the Russian Federation is low it has been argued that its socio-economic health determinants will need to be a major focus for improvement to create further gains in terms of health outcomes.^3^ However, to date, there has been no scrutiny of the Russian health system’s financial risk protection beyond established metrics such as CHE.

###  Background of the Healthcare System in the Russian Federation

 The cornerstone of the universal healthcare system in the Russian Federation is Federal Obligatory Medical Insurance (FOMIF), which was introduced in 1993. FOMIF is a mandatory health insurance funded by employer contributions.^4^ FOMIF represents the largest of three healthcare expenditure channels in Russia, although out-of-pocket (OOP) expenditure is also high, making up as much as 40% of total health expenditure.^5^ Employers’ contributions to FOMIF account for 5.1% of all wages. Those who are not employed are covered by regional (Oblast) budgets.^6^ Under FOMIF, Health Insurance Organizations were established to purchase medical care from providers and to incentivize high-quality care by increasing efficiency and responsiveness.^7^ Today, 99% of the population (employees and their dependents) are covered by FOMIF. The remaining 1% include prisoners and military personnel, who are covered by government programs with the same benefits package as FOMIF insurers.^6^

 There are several important characteristics of the FOMIF benefits package. First, it is quite comprehensive, covering outpatient and inpatient care, as well as tertiary and specialized services, with some of these being funded additionally from federal and regional budgets.^8^ Second, the benefits package is uniform across different population groups – working and non-working, poor and rich, more or less vulnerable^8^ – and there are no separate targeted health programs for the poor, arguably impacting their capacity to seek healthcare. Third, it is not always clear which specific diagnostic tests, medicines, implants, and curative procedures are provided within the benefits package as the scope of the benefits package has changed very little since its introduction in the early 2000s.^8^ While the majority of healthcare services are provided free of charge, there is a list of uncovered services, whose costs are paid OOP. This list includes pharmaceuticals for outpatients (except for exempt groups such as children, pensioners, and war veterans), cosmetic surgery, dental care (except for children, veterans, and other special groups), as well as medical prostheses, including dentures.^9^ Due to the negative list of services, roughly two-fifths of total healthcare expenditure in Russia is OOP, a value markedly smaller than in India, but comparable to other BRIC countries.^1^ OOP expenditure is higher for outpatient care; it even increased by 77% between 2005 and 2010.^10^

 The rollout of FOMIF was accompanied by significant increases in public spending on healthcare.^11^ However, public spending on healthcare, while being above the average for middle income countries, is still lower than the average for European Union countries.^11^ Within the federative regions of the Russian Federation, there has been some fiscal redistribution in order to improve resource allocation to less well-off regions and rural areas, but there are persistent spending variations across regions.^8^ Regional politics as well as socio-economic differences across regions are commonly identified as the main driving forces behind these persistent spending variations across regions.^12^

 Limited public funding, health system inefficiencies, and geographical disparities in health services supply continue to contribute to the high OOP spending. Moreover, low salaries for physicians and the cultural remnants of the previous healthcare system create an environment where informal payments thrive.^7,9^

###  Existing Literature on CHE and Unmet Need in the Context of the Russian Federation

 While there have been no attempts to scrutinize the financial risk protection of the Russian healthcare system, research in the wider European region offers a useful benchmark for such an analysis. For example, a recent study by the World Health Organization (WHO) finds a wide variation in CHE and the impoverishing effects of OOP across a sample of 24 European countries (Russia was not included). Moreover, when capacity to pay is used as a CHE metric, the study finds that CHE is consistently most heavily concentrated among the poorest fifth of the population and that the CHE is mainly driven by spending on outpatient medicines, followed by inpatient care and dental care.^13^ Furthermore, the study finds that in a few countries in the region, while the incidence of CHE is low, there is a high level of unmet need, particularly among poorer people, suggesting problems with healthcare affordability.^13^

 Existing studies suggest that, in Russia, a small share of the population is at risk of incurring CHE. According to a recent study, if CHE is defined as at least 10% of the household annual budget, only 5% of the population is at risk of incurring CHE; the share further falls to 0.6% if a more stringent threshold of 25% is used.^14^ Despite the low CHE risk, there is evidence of people forgoing needed healthcare. For example, based on nationally representative data gathered in 2001, Balabanova et al^15^ report that 11.3% of respondents had to forgo medical services frequently and 16.8% of respondents had to forgo such services sometimes. For pharmaceuticals, 16.8% of respondents were never able to obtain medication and 32.0% could not obtain them sometimes. In a follow-up study, Balabanova et al^16^ find that Russians are less likely to forgo healthcare, compared to citizens of the other countries that emerged from the former Soviet Union, echoing some of the findings from studies exclusively focusing on Russia.^10^ However, people in Russia, particularly the poor and rural populations, continue to forgo medicines, although the share of people forgoing medicines has decreased over time.^17^ While financial difficulty is the main reason for forgoing healthcare, Balabanova et al^15^ report additional reasons, including self-treatment, purchasing pharmaceuticals without obtaining a doctor’s prescription, long waiting times to see a health professional, and a lack of trust in staff qualifications. Furthermore, despite significant health sector investment, patients in Russia continue to describe the quality of care as poor, which, ultimately, impacts upon their decision to seek care.^18^ Patient dissatisfaction with health services is due to long waiting times and limited availability of modern medical equipment and medicines, as well as dissatisfaction with the availability and quality of medical personnel.^19^

 Against this backdrop, CHE can be observed only for households or individuals who seek healthcare. Therefore, although the share of people experiencing CHE is low, it might reflect barriers to accessing healthcare. In other words, as argued by the WHO study mentioned above, when healthcare is forgone, CHE fails to capture the full financial implications of the healthcare system.^13^ Similar to the WHO study, we assess the usefulness of focusing on CHE as the sole metric for assessing financial risk protection, and coupled it with an assessment of unmet need for healthcare. This is particularly relevant in Russia – a country with a low level of public healthcare spending. However, we go a step further than the WHO study, firstly, by extending our analysis to include the budget approach in measuring and defining CHE (in addition to the capacity-to-pay approach); and secondly by focusing on all forms of unmet need for healthcare (not only unmet need for dental and outpatient pharma products). Our study set three specific objectives: (*i*) to estimate CHE in Russia; (*ii*) to study the impoverishing effect of OOP expenditure; and (*iii*) to assess the extent of the unmet need.

## Methods

###  Data

 The Russia Longitudinal Monitoring Survey (RLMS) is a nationally representative survey series, including approximately 5000 households annually, which was established as a routine measure of health and economic status of households in the Russian Federation. Here, we focus on the period from 2010 to 2017. The surveys contain detailed information on both households (eg, level of income and consumption) as well as individuals (eg, healthcare utilization).^20^ Because the RLMS follows residences and not households, the survey has a non-random attrition rate.^21^ Hence, to ensure the representativeness of the survey, each round is replenished. Because of the methodological specificities, and to arrive at nationally representative estimates, we focused our analysis on repeated cross-sections of the RLMS.

 The RLMS sample is a multi-stage probability sample of dwellings. The response rate is relatively high and it exceeds 80%. Lower rates in Moscow and St. Petersburg were anticipated at the design stage, and initial allocations to these strata were increased to offset expected losses from refusal and noncontact.^20^ While in Supplementary file 1 (Table S1), we provide further information on the exact number of households that participated in each round, it is worth pointing out that in 2014 (and onwards) the sample size was cut by about 20%, because the cost of the project increased due to inflation. It should be stated that the procedures followed to cut the sample size guarantees that the smaller sample is still representative at the national level.^20^ In this study, we work with the cross-sectional sample of RLMS, which is representative of the entire Russian Federation. In addition and as a complementary exercise, we also use the longitudinal sample, which includes households that appeared in all rounds from 2010 until 2017. However, given the significant attrition rate we caution against viewing these findings as representative and we treat this exercise as a complementary to the one conducted on the cross-sectional sample. Information for household-level variables is provided by one member of the household, usually its most knowledgeable member (particularly on matters such as expenditure). This is an established practice, particularly for middle-income countries.^20^

###  Methodology

 We commenced our analysis by analyzing CHE across the entire sample as well as on a year-by-year basis. We then proceeded with an analysis of CHE by quintiles of socio-economic status (SES). SES quintiles were defined by the total household income per household member (equivalized) using the OECD equivalence scale, which assigns a value of 1 to the first household member, 0.7 to each additional adult and 0.5 to each child.^22^ In addition, we repeated the analysis using consumption as a proxy for SES (the results of this exercise are reported in Supplementary file 1). It is worth pointing out that 4% of income observations and 0.01% of consumption observations were missing, which does not, however, impact upon the validity of our results.^23^

 There are two approaches to defining and measuring CHE. The first approach (which is sometimes referred to as a basic approach and is commonly used in the health economics literature) entails relating OOP healthcare expenditure to total household income or consumption.^14,24^ Using this approach, the CHE headcount can be calculated as a share of households from the total sample, whose OOP health expenditures (health_i_) as a fraction of the total household expenditure (Exp_i_) are higher than a previously defined threshold (z). In other words, if the indicator (E) capturing CHE takes a value of 1 if health_i_/Exp_i_>z, and 0; otherwise, the total CHE headcount can be calculated as:


(1) $$H=1/N\sum_{i=1}^{N}E_{i},$$

where N is the sample size.

 This is the main approach that we followed in this study. In defining the CHE, we used four thresholds for CHE: 10%, 25%, 30%, and 40% of total household expenditure.^14^ An additional robustness check involved defining CHE in terms of healthcare expenditure relative to total income, while using the same thresholds listed above (10%, 25%, 30%, 40%).^14^

 In order to be comprehensive in our assessment of CHE, we coupled our main approach with the so-called capacity to pay or ability to pay approach, as used in the WHO European study.^24^ This approach takes basic needs into consideration before calculating CHE.^24^ The most common capacity-to-pay approach entails relating OOP healthcare expenditure to household budget, excluding expenditures on food. The proponents of this approach argue that applying this technique takes into account different patterns of spending of the bottom 20% versus the top 20% of the population and hence it is better positioned to capture the real extent of CHE (particularly across the socio-economic gradient). In other words, as households progress up the socio-economic ladder, spending on necessities takes up a smaller share of the household budget, which makes paying for healthcare easier.^25^ Most recently and in the European context, the capacity-to-pay approach has been modified to also include spending on rent and utilities.^25,26^ When applying this metric in the Russian context, the results should be treated with care: 91% of the residences in the Russian Federation (as per RLMS) are owned, which renders the WHO metric very similar to the main capacity-to-pay approach. Nevertheless, to add further depth and robustness to our findings, and building on the work conducted by the WHO, we employed the two additional CHE metrics: (*i*) OOP healthcare expenditure as a share of total household budget, excluding expenditures on food (using a threshold of 40%^25^); and (*ii*) OOP healthcare expenditure as a share of total household budget, excluding expenditures on food, rent and utilities (using a threshold of 40%^25^).

 Furthermore, in addition to the incidence of OOP (ie, CHE), and as a complementary exercise, we also derived the intensity (overshoot) of CHE, which measures the average extent by which the OOP expenditure exceeds the corresponding CHE threshold.^27,28^ The overshoot is given by the following expression:


(2) $$O=1/N\sum_{i=1}^{N}Overshoot_{i},$$

where Overhoot_i_ = E_i_ X ((health_i_/Exp_i_)-z). Simply put, Overshoot_i_ is the difference between the health payments budget share and the predefined threshold for each household *i*. Finally, mean positive overshoot, which we also derived, is simply given by the ratio between overshoot and headcount. This measure demonstrates the average OOP expenditure among the households that exceeded the predefined threshold of CHE.^28^ As in the case of CHE, here as well we present the findings for the entire sample, followed by disaggregated analysis based on SES.

 In addition to examining CHE, we also estimated the impoverishing effect of OOP healthcare payments.^24^ In this analysis, we began by measuring the impoverishing effect of OOP healthcare expenditure using the poverty headcount as a principal indicator of analysis. If Exp_i_ is the total consumption expenditure of household (i), the poverty headcount gross of healthcare payment (Hg) is:


(3) $$Hg\;=\;\Sigma \;G_{i},\;i=1\;to\;N,$$

where G_i_ = 1 if [(Exp_i_ /hhsize_i_ <PL] and 0 otherwise, hhsize_i_ is the household size, N is the number of households in the sample, while PL the poverty threshold.

 The poverty headcount net of healthcare payments (Hn) can be calculated as:


(4) $$Hn\;=\;\Sigma \;J_{i},\;i=1\;to\;N,$$

where J_i_ =1 if [(Exp_i_ – health_i_)/hhsize_i_< PL], with health_i_ being the household’s OOP healthcare expenditure. The difference between Hn and Hg provides the impoverishing effect of healthcare payments.

 In addition, we coupled this with an analysis of the impoverishing effect of OOP using two additional indicators: the poverty gap and the normalized poverty gap. The poverty gap is the average amount by which resources fall short of the poverty line, while the normalized poverty gap is obtained by dividing the poverty gap by the poverty line.^29^ In assessing the impoverishing effect of OOP, household equivalized scales were used in order to express healthcare expenditures per adult equivalent household member. Nominal gross and net healthcare payments were expressed in constant 2011 terms using the Consumer Price Index,^30^ and then converted to US dollar, using the purchasing power parity (PPP) conversion factors.^1^ By doing this, the impoverishing effect could be assessed against the three widely used poverty thresholds: US$1.9 per day, US$3.2 per day and US$5.5 per day, all three in 2011 prices, PPP, consistent with established practice.^24^

 Next, and building on the WHO juxtaposition of CHE and forgoing healthcare, we conducted an analysis of unmet healthcare need.^13^ Households were considered to have forgone healthcare if they answered affirmatively to the question, “In the last twelve months, were you or a member of your household unable to access the following healthcare services because of financial difficulty: (*a*) inpatient care, (*b*) outpatient care, (*c*) medications, and (*d*) dental care?” Unlike the WHO study, which focused only on unmet need for dental services and outpatient medications, in our study we also analyzed unmet need for inpatient and outpatient care, which, arguably account for the largest share of healthcare services. As a complementary analysis, we constructed an additional measure of unmet need which takes into account two conditions: if households have reported forgoing healthcare and if they, simultaneously, have incurred zero healthcare expenditure. By combining these two measures, we captured the extent to which unmet need entirely prevents households from using healthcare.

 All of the analyses above were conducted based on questions included in the household module of the survey, for the following reasons. First, as further described by the survey, while, ideally, the individual survey should be administered to all members of the household, in practice, the response rate among the elderly and children (who also happen to be the biggest users of healthcare services) is much lower, which would further limit an individual level analysis.^20^ Second, the information on unmet need is asked at household level, and thus, in order to compare likes with likes, we have also based the analysis on the household module. Third, it is standard practice, as established by the WHO and World Bank, to use household level information when analyzing the financial risk protection of a healthcare system.^2,13^ Hence, we leave the individual level analysis for further future exploration.

 All our analyses were conducted using Stata 14 and we used the nationally representative weights provided in the survey.

###  Limitations

 Our study has certain limitations. First, we established correlation by our analysis of CHE and the impoverishing effect of OOP expenditure, rather than causation between SES and the variables of interest. Second, as mentioned above, the structure of the RLMS questionnaire does not allow direct replication of the results of Wagstaff et al (in other words, we do not take into account utilization of healthcare).^2^ Third, the survey is not representative at sub-national (ie, Federal District level or further down to Oblast level); hence, it was not possible to do a sub-national analysis, which would also take into account supply-side effects (eg, availability of healthcare infrastructure). Finally, RLMS does not include information on additional barriers to access (thus including paying for healthcare informally), such as availability of transport to healthcare facilities, which could shed light on other reasons for not seeking needed care.

## Results

Table 1 captures the results of CHE on the entire sample as well as on a year-by-year basis. There are a few results that stem from this analysis. First, it is evident that the share of households that incur CHE reduces as the CHE threshold increases. For example, when considering the entire sample, 7.7% of households incurred CHE considering the 10% threshold, 1.59% when considering the 25% threshold, 1.07% when considering the 30% threshold and finally, 0.49% when considering the 40% threshold. The analysis conducted on a year-by-year basis yields another important finding: as Russia entered a period of economic slowdown (from 2014 onwards), the share of households that incurred CHE slightly decreased across all CHE thresholds used in this analysis, potentially suggesting that reduction in healthcare expenditure was one of the coping mechanisms used during the period of economic distress.

**Table 1:** Percentage Share of Households With CHE (Measured as a Share of Total Household Consumption), Pooled RLMS Data, 2010-2017

	**10% Threshold**	**25% Threshold**	**30% Threshold**	**40% Threshold**
Entire sample	7.7	1.59	1.07	0.49
2010	7.74	1.74	1.24	0.57
2011	8.58	1.96	1.38	0.61
2012	9.11	1.97	1.33	0.64
2013	8.81	2.01	1.24	0.60
2014	8.18	1.70	1.13	0.54
2015	6.53	1.26	0.91	0.47
2016	5.68	1.02	0.60	0.21
2017	5.88	0.67	0.43	0.14

Abbreviations: CHE, catastrophic healthcare expenditure; RLMS, Russia Longitudinal Monitoring Survey. Source/Notes: RLMS.

 Figure captures CHE by income quintile, using four separate thresholds of 10%, 25%, 30%, and 40%, respectively. There are a few observations that stem from the data. First, as expected, the share of households incurring CHE decreased as the CHE threshold increased. Second, the share of households incurring CHE increased among higher SES households (for the 10% threshold, it reached a plateau for the second and third income quintiles before decreasing slightly for the fourth and fifth ones). For example, for the bottom quintile, the share of households incurring CHE amounted to 6.04% when considering the 10% threshold, 0.95% when considering the 25% threshold, 0.65% when considering the 30% threshold, and 0.33% when considering the 40% threshold. The share of households incurring CHE in the top SES quintile was higher across all thresholds: 7.35%, 1.92%, 1.35%, and 0.68%, respectively. More importantly, a χ^2^ test suggest a strong correlation between SES and CHE (Pearson χ^2^ = 55.548 (*P* = .000) for 10% threshold, Pearson χ^2^ = 29.396 (*P* = .000) for 25%, Pearson χ^2^ = 21.71 (*P* = .000) for 30% and Pearson χ^2^ = 15.467 (*P* = .000) for the 40% threshold). These results are consistent when the analysis was conducted on the separate waves of the survey. Moreover, the results are broadly consistent when using consumption as a proxy for SES, with the caveat that the lowest consumption quintile exhibits higher CHE (see Figure S1 of Supplementary file 1 for details). In addition, we also present the distribution of households according to their share of healthcare expenditure (see Figure S2 of Supplementary file 2). As evidenced by the figure, an overwhelming majority of households (about four fifths) devote between 0% and 5% of their total household expenditures on healthcare.

**Figure:**
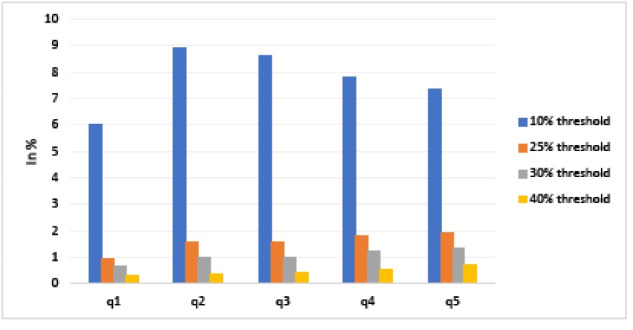
Percentage Share of Households With CHE (Measured as a Share of Total Household Consumption) and Assessed Against the Relevant Threshold, Per Income Quintile, Pooled RLMS Data, 2010–2017.Abbreviations: CHE, catastrophic healthcare expenditure; RLMS, Russia Longitudinal Monitoring Survey. Source/Notes: RLMS.

 Further, not only CHE but also total OOP healthcare spending increased with SES. The analysis shows that the top two income quintiles accounted for 58.2% of the total OOP spending, compared to 24% for the bottom two. Furthermore, the gap was even wider when conducting the analysis based on consumption quintiles: the top two quintiles accounted for the 65.2% of the total OOP healthcare spending in the country, compared to 18.7% for the bottom two. This analysis further illustrated that OOP expenditure was mostly generated by higher income households. In addition to the exercises above, we have also conducted an analysis of the main drivers of CHE. According to our analysis, when considering the CHE threshold of 10%, an overwhelming share of CHE expenditure (58%) is driven by spending on medications. The share drops to 37% when raising the CHE threshold to 25%, but still, the expenditures on medications represent the main drivers of CHE. When considering higher thresholds (of 30% and 40%), spending on dental care is the main driver of CHE, followed by medications.

 The results above are consistent regardless of the definition for CHE used, ie, OOP healthcare expenditure as a share of total consumption excluding food, OOP healthcare expenditure as a share of consumption excluding food, rent and utilities or OOP healthcare expenditure as a share of income (eg, Supplementary file 1, Figures S3 and S4). When using income in the denominator, the share of households with CHE was slightly higher; however, this is likely to be the result of the under-reporting of income in RLMS, which has previously been documented.^31^

 As a complementary exercise, we have repeated the analysis above on a subset of households appearing in all rounds (2010-2017), ie, the longitudinal sample. The findings of this analysis which are reported in a separate section of Supplementary file 2 (Table S5 and Figure S7-S11) closely match the findings reported for the cross-sectional sample described above.

 As mentioned in the methods section, we couple the analysis incidence of OOP (ie, CHE) with an analysis of intensity of catastrophic costs (overshoot and mean positive overshoot). The results are provided for the entire sample and on year-by-year basis in Table S2 (Supplementary file 1). The findings suggest that not only is the incidence of CHE low, but so is its intensity. Moreover, the findings on intensity of catastrophic costs by SES indicate that intensity of catastrophic costs increases with SES, closely mimicking the findings on CHE incidence (Table S3, Supplementary file 1). As in the case of CHE incidence, we complement this analysis with an analysis of the longitudinal sample (Supplementary file 2, Table S6 and S7) and they closely match the findings established for the cross-sectional sample.

Table 2 captures the impoverishing effect of OOP by using three different thresholds for the poverty headcount and poverty gap: US$1.9, US$3.2, and US$5.5 (all three in 2011 US dollar, PPP), while Table S4 in Supplementary file 1 reports the results on a year-by-year basis.^32^ As explained in the Methods section, the impoverishing effect of OOP captured the difference of the poverty headcount ratio gross and net of healthcare payments. Overall, the poverty headcount ratio gross of healthcare payments was low (0.2% using the US$1.9 per day threshold, less than 0.5% using the US$3.2 per day threshold, and about 1.3% when using the US$5.5 per day threshold). Similarly, the poverty headcount ratio net of healthcare payments was low. Hence, the impoverishing effect of OOP healthcare payments was negligible when considering all three poverty thresholds. The results on a year-by-year basis (Table S4, Supplementary file 1) show that the impoverishing effects of OOP are small and, moreover, they remained small during the most recent economic slowdown (2014-2016). Furthermore, Table 2 and Table S4 (Supplementary file 1) also present the results of additional two indicators (poverty gap and normalized poverty gap) and they reconfirm the findings that the impoverishing effect of OOP in Russia is low (both when the entire sample is analyzed and when the analysis is conducted on a year-by-year basis). Finally, these results are comparable to the ones arrived at for the longitudinal sample (Supplementary file 2, Table S8 and S9).

**Table 2:** Impoverishing Effects of OOP (Poverty Headcount, Poverty Gap and Normalized Poverty Gap), (in %), Pooled RLMS, 2010-2017

**Poverty Headcount Gross of Healthcare Payments**				**Poverty Headcount Net of Healthcare Payments**				**Difference**			
	**1.9 USD Per Day, Constant 2011, PPP**	**3.2 Per Day, Constant 2011, PPP**	**5.5 USD Per Day, Constant 2011, PPP**		**1.9 USD Per Day, Constant 2011, PPP**	**3.2 Per Day, Constant 2011, PPP**	**5.5 USD Per Day, Constant 2011, PPP**		**1.9 USD Per Day, Constant 2011, PPP**	**3.2 Per Day, Constant 2011, PPP**	**5.5 USD Per Day, Constant 2011, PPP**
Poverty headcount	0.2	0.5	1.3	Poverty headcount	0.3	0.6	1.6	Poverty headcount	0.0	0.1	0.3
Poverty gap	0.9	1.8	3.5	Poverty gap	0.9	1.8	3.5	Poverty gap	0.0	0.0	0.0
Normalized poverty gap	0.5	0.6	0.6	Normalized poverty gap	0.5	0.6	0.6	Normalized poverty gap	0.0	0.0	0.0

Abbreviations: OOP, out-of-pocket; RLMS, Russia Longitudinal Monitoring Survey; PPP, purchasing power parity. Source/Notes: RLMS.

 The final set of analyses we perform in this paper relates to unmet need. As mentioned in the methodology of the paper, we commence the analysis by reporting on households who have forgone different types of healthcare (Table 3). When considering the total sample, the largest share of households have forgone dental care (10.85%) followed by medications (7.8%), inpatient care (4.21%) and then outpatient care (3.6%). We also observe that as the effects of the economic slowdown started to be felt (between 2014 and 2016), there is a gradual increase in the share of households who reported unmet need for all types of healthcare.

**Table 3:** Percentage Share of Households With Unmet Need (as Reported in the Survey), by Type of Healthcare Service, Pooled RLMS Data, 2010-2017

	**Unmet Dental Care**	**Unmet Pharmaceutical Care**	**Unmet Inpatient Care**	**Unmet Outpatient Care**
Entire sample	10.85	7.77	4.21	3.60
2010	12.07	7.64	4.29	4.27
2011	9.41	6.94	3.55	2.97
2013	13.10	11.46	6.52	4.54
2014	9.72	6.92	3.41	3.12
2015	10.34	7.19	3.66	2.92
2016	11.07	7.21	3.80	3.41
2017	9.80	6.54	3.95	3.78

Abbreviation: RLMS, Russia Longitudinal Monitoring Survey. Source/Notes: RLMS.

 Furthermore, the supplementary materials report the disaggregated analysis by type of unmet need (dental, medications, inpatient, and outpatient care) and by SES. The results follow the SES patterns above in that unmet need is concentrated among the lowest socio-economic strata and, furthermore, unmet need is concentrated in dental services, followed by medications, and inpatient and outpatient care (Supplementary file 1, Figure S5). In addition, and as mentioned in the methodology section, we conducted an additional exercise where we accounted for households that simultaneously experience unmet need and incur zero healthcare expenditure. We juxtaposed this exercise against households that report unmet need for medicines and certain services, even though they have used healthcare. These findings are reported in the Supplementary file 1 (Figure S6) and suggest that the share of households with unmet need (who simultaneously incur zero healthcare expenditure) and households with unmet need for medicines and certain services is higher in the lowest SES. Finally and as in the cases above, we repeated our analysis on the longitudinal sample, where the results closely match the ones obtained on the cross-section sample (Supplementary file 2, Table S9 and Figure S12 and S13).

## Discussion

 Using four thresholds (10%, 25%, 30%, and 40% of total household expenditure), as well as using definitions including both total budget (captured by consumption or income) and capacity to pay, we found that a small fraction of households incurred CHE in Russia. Moreover, the results using complementary measures (eg, overshoot of CHE) are consistent with our main finding for high financial risk protection within the Russian healthcare system. Our findings support those of Wagstaff et al,^14^ who find that CHE in Russia is low (amounting to 5% when using the 10% threshold and 0.6% when using the 25% one), and lower than the figure derived in our study. These differences may be attributable to the data sources: while we relied on the RLMS, Wagstaff et al^14^ used the annual household budget survey conducted by the national statistics office. There are some differences in the surveys such as periodicity (RLMS is yearly whereas Household Budget Survey is quarterly), which most likely has an impact on the final results^33^. In addition, the findings of both, our study and Wagstaff et al^14^ put Russia in a similar group of high and upper-middle income countries in the wider European region.^13^

 Second, we found the impoverishing effect of OOP expenditure to be small. This is not surprising given recent evidence which suggests that the impoverishing effect of OOP usually occurs in low income countries.^34^ The rates of poverty slightly increased during the most recent slowdown (2014-2016), although the overall impoverishing effect of OOP remained limited. The recent slowdown was accompanied by a sharp depreciation of the ruble, pushing hundreds of households below the poverty line.^35^ In other words, the near-poor and vulnerable may be less able to use protective measures during periods of economic distress, resulting in an increase in the poverty rate.^36^ Conversely, the impoverishing effect of OOP remained limited, which could be explained by a noticeable reduction in CHE during the crisis years. This implies that the shrinking of OOP payments on healthcare occurred at a faster pace than the shrinking of domestic budgets. Put simply, the reduction of CHE during the 2014-2016 slowdown was an artifact of the crisis: postponing using healthcare (and thus OOP expenditure) could have been adopted as a coping mechanism, particularly among households heavily affected by the crisis. Our findings using the metrics of intensity of CHE (overshoot of CHE) reconfirm this notion. Previous findings suggest that some of those at risk of poverty may reduce healthcare utilization to prevent impoverishment.^37^ Our finding thus further suggests that the analysis of CHE as a metric of financial risk protection should be coupled with an analysis of unmet need, particularly in periods of economic distress.

 Third, our analysis shows that despite the low CHE level, many forwent seeking care, resulting in overall unmet need, or unmet need for medicines and certain services. In particular, the results of the disaggregated analysis show that most unmet need was concentrated in dental services and medications. This finding echoes previous findings in the wider European region^13^ and it is due to two reasons. First, there were significant gaps in coverage for dental healthcare services and medications, particularly for outpatients.^9^ A recent report by the World Bank argues that OOP expenditure is dominated by outpatient drugs, which are not adequately covered by the state-guaranteed benefits package and could potentially lead households to incur CHE.^8,38^ While only 10% of the Russian population has sufficient outpatient drug coverage, the rest are paying OOP, similarly to other middle income countries.^8,39^ Hence, poorer people may be forced to forgo purchasing needed medications. Second, some of the forgone care could be attributed to geographical supply-side disparities and informal healthcare payments.^40-42^ Individuals who cannot afford informal payments may thus forgo the necessary healthcare.^43^ Furthermore, while government programs have sought to improve the supply of doctors in rural areas, rural patients continue to face non-financial barriers to seeking care, such as a lack of doctors or long waiting times.^44^ Broadly speaking, excessive waiting times persist^45^ as a common form of healthcare rationing driven by lower-than-needed public spending. In the long run, however, the literature suggests that delaying seeking care today is associated with worse health outcomes, longer periods of hospitalization, poorer prognoses, and increased psychological distress, ultimately increasing OOP payments as well as increasing the risk of CHE.^46-49^

 Fourth, building on the WHO approach, we applied two additional CHE metrics based on the concept of capacity to pay, in order to study the distributional impact of CHE.^13^ We did this given the established practice of defining and calculating CHE in the wider European region.^13^ While our results are robust when these additional metrics are applied, they should be used with care. First, we caution against a blanket approach to defining “basic necessities” even in the wider European context. Indeed, as 91% of the residences in Russia (as per the RLMS) are owned, the two capacity-to-pay metrics used in this study are similar to each other. Second, and more broadly, there is growing evidence to support the questioning of the usefulness of these measures when calculating CHE. As recently argued by Wagstaff et al,^24^ while, at first sight, the capacity-to-pay measure might be a good complement to the basic approach to CHE, it falls short of showing how far OPP spending eats into resources required for necessities, and therefore does not show how far such spending leads to absolute hardship nor how close this is to happening.^24^ In other words, as Wagstaff et al go on to document, if one is to show the relative hardship associated with OOP, one should express OOP relative to consumption (or income) and not capacity to pay; by contrast, if the goal is to show absolute hardship associated with OOP, one should apply the concept of impoverishment, as we do in this study.^24^

## Conclusion

 Using eight waves of the RLMS, we found evidence of low CHE levels across wealth quintiles in Russia. More specifically, our findings suggest that in countries with significant unmet healthcare need due to financial barriers or supply-side inequalities, such as Russia, an analysis of CHE (and related measures) cannot fully capture the inefficacies in the health system’s financial protection and it should be coupled with a study of unmet need. Some of the shortcomings of CHE, as demonstrated by our analysis, were further exacerbated during periods of economic distress. Furthermore, our study suggests that the impoverishing effect of OOP payments in the Russian Federation was still low and remained limited during the most recent economic downturn. A further scrutiny of unmet need revealed that some households reported unmet need, particularly for medications and dental care. This finding further illustrated the need for a joint assessment of CHE and unmet need. In addition, and consistent with the broader literature, these findings also demonstrate the existing inequities in the healthcare system.^50,51^ Consequently, further policy interventions should target financial barriers to seeking care, particularly for the poor and vulnerable. More specifically, increasing coverage for pharmaceuticals and dental care could have an impact on the overall reduction of OOP payments, as well as reducing the unmet need for medications and dental care.^52^

## Acknowledgements

 We are grateful to the Higher School of Economics for making the microdata available for analysis.

## Ethical issues

 As this is a secondary data analysis, no ethical clearance was needed.

## Competing interests

 Authors declare that they have no competing interests.

## Authors’ contributions

 ZN and EM designed the study. ZN conducted that data analyses with inputs and verification from JC. All authors drafted the final version of the manuscript.

## Supplementary files

Supplementary file 1.Additional Results Based on the Cross-sectional Sample.

Supplementary file 2. Additional Results Based on the Longitudinal Sample.
