# Supplementary material for: Financial Risk Protection and Unmet Healthcare Need in Russia
Source: Int J Health Policy Manag. 2021 Jul 14;11(9):1715–24. doi: 10.34172/ijhpm.2021.72 (PMC9808245; doi:10.34172/ijhpm.2021.72)
Supplement: Supplementary file 1 — Additional Results Based on the Cross-sectional Sample. [file ijhpm-11-1715-s001.pdf]

Article title: Financial Risk Protection and Unmet Healthcare Need in Russia

Journal name: International Journal of Health Policy and Management (IJHPM)

Authors' information: Zlatko Nikoloski*, Jane Cheatley, Elias Mossialos

Department of Health Policy, London School of Economics and Political Science, London, UK.

(*Corresponding author: z.nikoloski@lse.ac.uk)

Supplementary file 1. Additional Results Based on the Cross-sectional Sample

Table S1. Number of households (per round) in the cross-sectional and longitudinal sample

year	Cross-sectional sample	Longitudinal sample
2010	6325	3221
2011	6385	3221
2012	6516	3221
2013	6148	3221
2014	4872	3221
2015	4862	3221
2016	4849	3221
2017	4855	3221

Source/Notes: RLMS

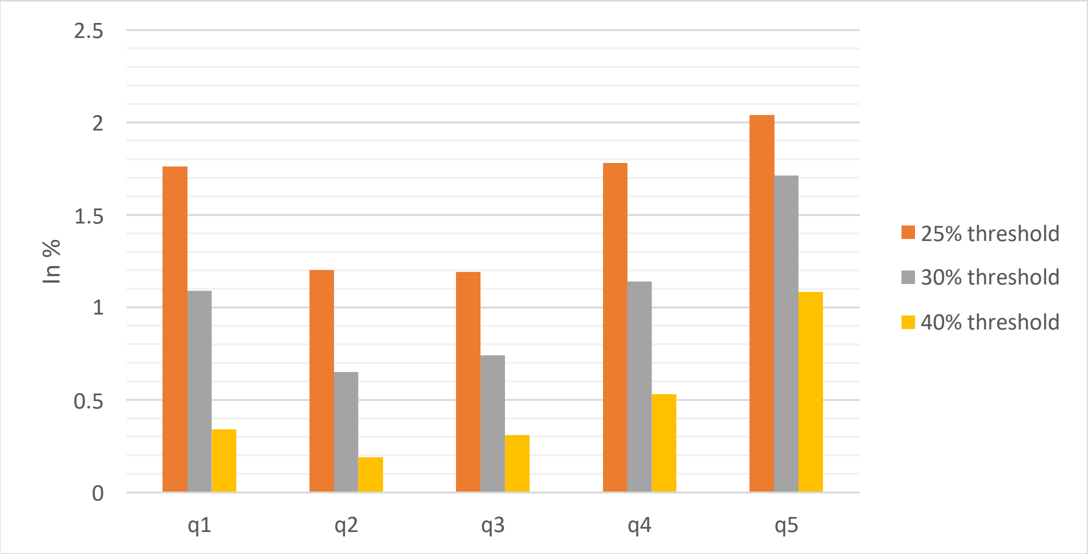

Figure S1. Percentage share of households with catastrophic health care expenditure (measured as a share of total household expenditure) and assessed against the relevant threshold, per consumption quintile, pooled RLMS data 2010–2017.

Source/Notes: RLMS. The following values for the Pearson coefficient are reported: 25% threshold – Pearson chi2=30.206 (p=0.000), 30% - Pearson chi2=53.913 (p=0.000), 40% - Pearson chi2=82.74 (p=0.000)

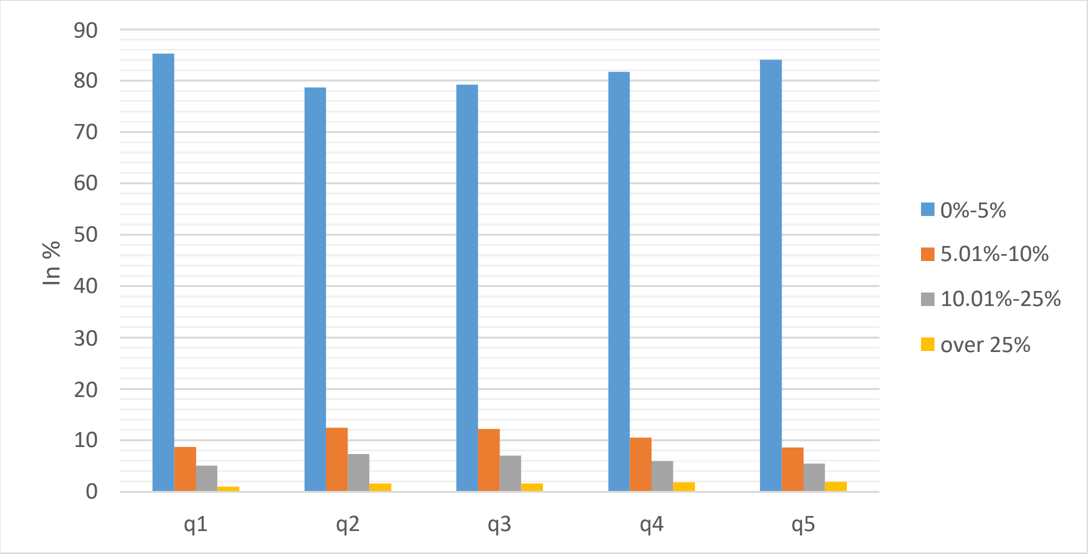

Figure S2. Distribution of households with respective expenditure on healthcare (as a share of total consumption), by income quintiles (in %), pooled RLMS data 2010–2017.

Source/Notes: RLMS. The following value for the Pearson coefficient are reported – Pearson chi2=211.487 (p=0.000).

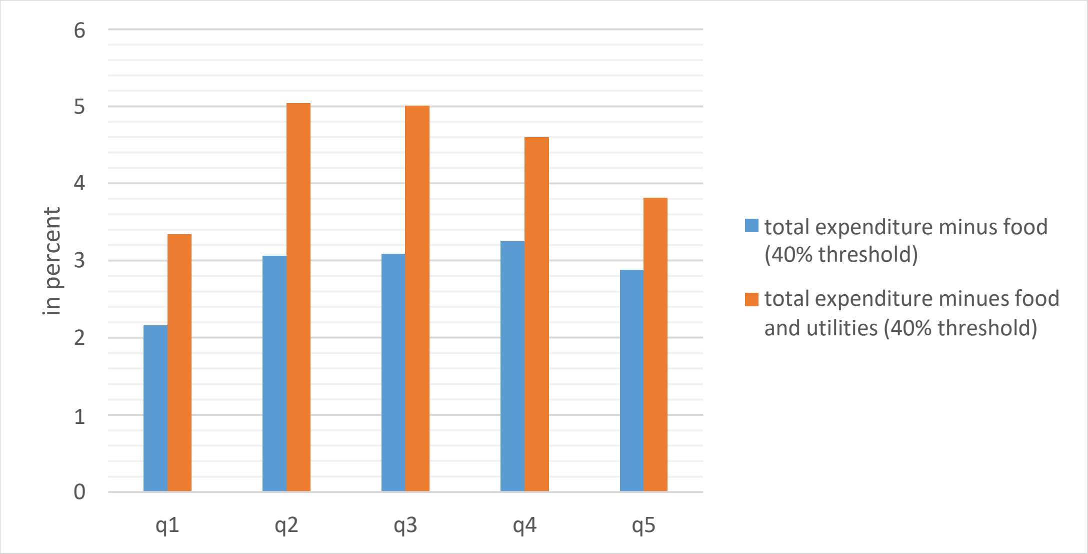

Figure S3. Percentage share of households with catastrophic health care expenditure (measured as a share of healthcare expenditure in total expenditure minus food and total expenditure minus food, rent and utilities), per income quintile, pooled RLMS data 2010–2017.

Source/Notes: RLMS. The following values for the Pearson coefficient were obtained. When using 40% threshold of total expenditure less food, Pearson chi2=43.44 (p=0.000), while when using 40% threshold of total expenditure less food and utilities, Pearson chi2=20.048 (p=0.000)

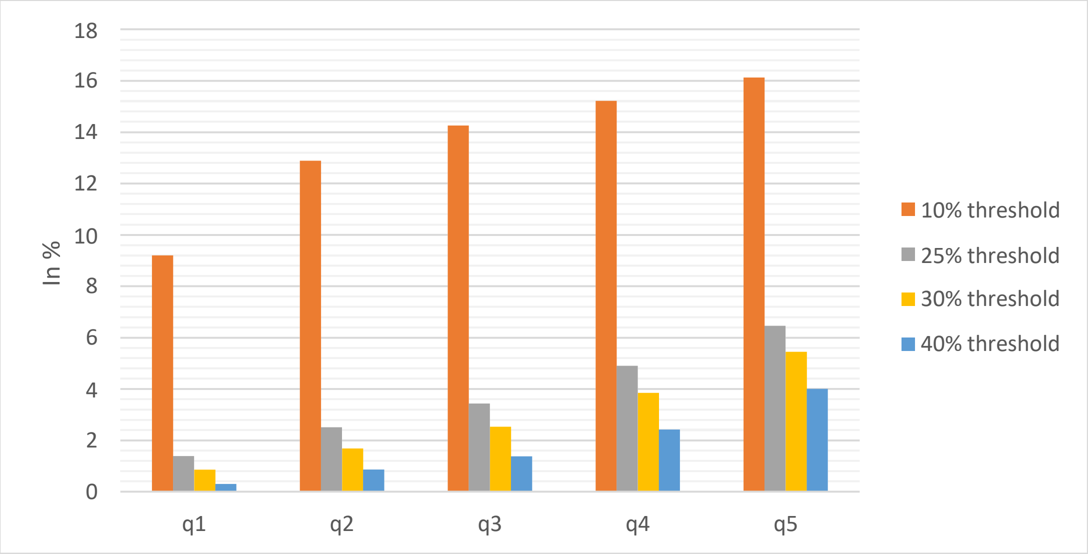

Figure S4. Percentage share of households with catastrophic health care expenditure (as a share of income), per consumption quintile, pooled RLMS data 2010–2017.

Source/Notes: RLMS. The following values for the Pearson coefficient were obtained on the link between SES and CHE. When using the 10% threshold, Pearson chi2=209.67 (p=0.000), when using 25%, Pearson chi2=361.877 (p=0.000), when using 30% threshold, Pearson chi2=386.235 (p=0.000) and when using the 40% threshold, Pearson chi2=386.69 (p=0.000)

Table S2. Overshoot and mean positive overshoot of the CHE (measured as a share of total household consumption), (in %), pooled RLMS, 2010-2017

	10% threshold	25% threshold	30% threshold	40% threshold
Overshoot	0.74	0.2	0.13	0.06
Mean positive overshoot	9.6	12.65	12.74	12.38
Overshoot by year				
	10% threshold	25% threshold	30% threshold	40% threshold
Overshoot - 2010	0.8	0.25	0.18	0.09
Mean positive overshoot - 2010	10.47	14.83	14.89	16.52
Overshoot - 2011	0.89	0.26	0.17	0.084
Mean positive overshoot - 2011	10.38	13.4	12.98	12.79
Overshoot - 2012	0.94	0.28	0.2	0.09
Mean positive overshoot - 2012	10.39	14.28	14.98	15.34
Overshoot - 2013	0.86	0.23	0.15	0.06
Mean positive overshoot - 2013	9.87	11.58	12.34	10.46
Overshoot - 2014	0.8	0.2	0.13	0.055
Mean positive overshoot - 2014	9.84	11.97	11.75	10.3
Overshoot - 2015	0.6	0.15	0.1	0.04
Mean positive overshoot - 2015	9.17	12.09	10.9	8.01
Overshoot - 2016	0.46	0.08	0.045	0.01
Mean positive overshoot - 2016	8.26	8.05	7.62	4.83
Overshoot - 2017	0.38	0.06	0.04	0.01
Mean positive overshoot - 2017	6.6	9.9	9.31	8.76

Source/Notes: RLMS

Table S3. Overshoot and mean positive overshoot of the CHE (measured as a share of total household consumption) per income quintile, (in %), pooled RLMS, 2010-2017

	10% threshold	25% threshold	30% threshold	40% threshold
Overshoot q1	0.5	0.1	0.07	0.02
Mean positive overshoot – q1	8.4	11.98	11.21	8.03
Overshoot - q2	0.7	0.1	0.12	0.06
Mean positive overshoot – q2	8.5	12.14	12.81	15.97
Overshoot - q3	0.7	0.2	0.12	0.06
Mean positive overshoot – q3	8.85	11.45	11.94	12.9
Overshoot - q4	0.8	0.23	0.15	0.07
Mean positive overshoot – q4	10.48	12.83	12.83	12.1
Overshoot - q5	0.8	0.26	0.17	0.08
Mean positive overshoot – q5	11.48	13.57	12.69	11.91

Source/Notes: RLMS. The following values for the Pearson chi2 were reported: for the link between SES and 10% overshoot- Pearson chi2=54.19 (p=0.000), SES and 25% overshoot – Pearson chi2=25.35 (p=0.000), SES and 30% overshoot – Pearson chi2=17.55 (p=0.000), SES and 40% overshoot – Pearson chi2=11.1 (p=0.025)

Table S4. Impoverishing effects of OOP (poverty headcount, poverty gap and normalized poverty gap), (in %), pooled RLMS, 2010-2017

	Gross of healthcare payments						Net of healthcare payments							difference		
		1.9 USD per day, constant 2011, PPP	3.2 per day, constant 2011, PPP	5.5 USD per day, constant 2011, PPP				1.9 USD per day, constant 2011, PPP	3.2 per day, constant 2011, PPP	5.5 USD per day, constant 2011, PPP				1.9 USD per day, constant 2011, PPP	3.2 per day, constant 2011, PPP	5.5 USD per day, constant 2011, PPP
2010	Poverty headcount	0.3	0.6	1.2		2010	Poverty headcount	0.3	0.6	1.4		2010	Poverty headcount	0.0	0.0	0.2
	Poverty gap	1.1	1.8	3.3			Poverty gap	1.1	1.8	3.3			Poverty gap	0.0	0.0	0.1
	Normalized poverty gap	0.6	0.6	0.6			Normalized poverty gap	0.6	0.6	0.6			Normalized poverty gap	0.0	0.0	0.0
2011	Poverty headcount	0.2	0.6	1.2		2011	Poverty headcount	0.2	0.6	1.4		2011	Poverty headcount	0.0	0.0	0.1
	Poverty gap	0.8	1.9	3.3			Poverty gap	0.9	1.9	3.4			Poverty gap	0.1	0.0	0.1
	Normalized poverty gap	0.4	0.6	0.6			Normalized poverty gap	0.5	0.6	0.6			Normalized poverty gap	0.1	0.0	0.0
2012	Poverty headcount	0.2	0.4	1.2		2012	Poverty headcount	0.2	0.5	1.5		2012	Poverty headcount	0.0	0.1	0.2
	Poverty gap	0.8	1.8	3.6			Poverty gap	0.8	1.8	3.6			Poverty gap	0.0	0.0	0.0
	Normalized poverty gap	0.4	0.6	0.7			Normalized poverty gap	0.4	0.6	0.7			Normalized poverty gap	0.0	0.0	0.0

2013	Poverty headcount	0.2	0.5	0.9		2013	Poverty headcount	0.2	0.5	1.0		2013	Poverty headcount	0.0	0.1	0.1
	Poverty gap	0.8	1.8	3.0			Poverty gap	0.8	1.8	3.0			Poverty gap	0.0	0.0	0.0
	Normalized poverty gap	0.4	0.5	0.5			Normalized poverty gap	0.4	0.5	0.5			Normalized poverty gap	0.0	0.0	0.0
2014	Poverty headcount	0.1	0.2	0.7		2014	Poverty headcount	0.2	0.3	0.9		2014	Poverty headcount	0.0	0.1	0.3
	Poverty gap	0.7	1.5	3.3			Poverty gap	0.7	1.7	3.4			Poverty gap	0.0	0.2	0.2
	Normalized poverty gap	0.4	0.5	0.6			Normalized poverty gap	0.4	0.5	0.6			Normalized poverty gap	0.0	0.1	0.0
2015	Poverty headcount	0.3	0.6	1.7		2015	Poverty headcount	0.4	0.6	2.0		2015	Poverty headcount	0.1	0.1	0.3
	Poverty gap	1.0	1.7	3.5			Poverty gap	1.0	1.7	3.5			Poverty gap	0.0	0.0	0.0
	Normalized poverty gap	0.5	0.5	0.6			Normalized poverty gap	0.5	0.5	0.6			Normalized poverty gap	0.0	0.0	0.0
2016	Poverty headcount	0.4	0.7	2.3		2016	Poverty headcount	0.4	0.8	2.7		2016	Poverty headcount	0.0	0.1	0.4
	Poverty gap	0.9	1.6	3.7			Poverty gap	0.9	1.8	3.7			Poverty gap	0.0	0.1	0.0

	Normalized poverty gap	0.5	0.5	0.7			Normalized poverty gap	0.5	0.5	0.7			Normalized poverty gap	0.0	0.0	0.0
2017	Poverty headcount	0.1	0.4	1.6		2017	Poverty headcount	0.1	0.5	1.9		2017	Poverty headcount	0.0	0.1	0.4
	Poverty gap	0.9	2.1	3.9			Poverty gap	0.9	2.2	3.9			Poverty gap	0.0	0.0	0.1
	Normalized poverty gap	0.5	0.7	0.7			Normalized poverty gap	0.5	0.7	0.7			Normalized poverty gap	0.0	0.0	0.0

Source/Notes: RLMS

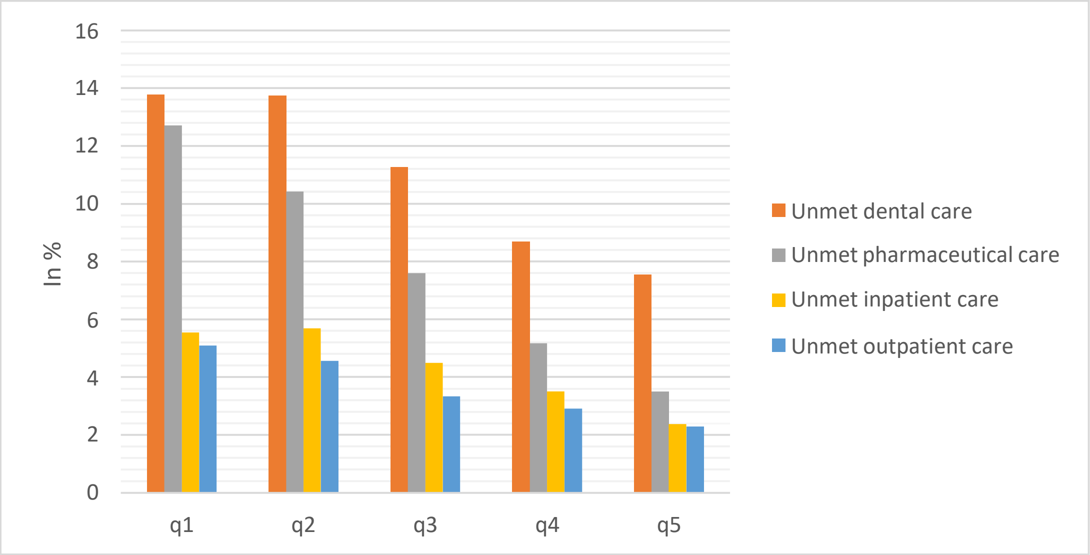

Figure S5. Percentage share of households with unmet need (as reported by the survey respondents), by income quintile and type of unmet need, pooled RLMS data 2010–2017

Source/Notes: RLMS. The following values for the Pearson chi2 are reported. In case of unmet dental care and SES, Pearson chi2=172.07 (p=0.000), unmet need for medicines and SES, Pearson chi2=497.491 (p=0.000); unmet need for inpatient care and SES, Pearson chi2=86.49 (p=0.000); unmet need for outpatient care and SES, Pearson chi2=84.29 (p=0.000)

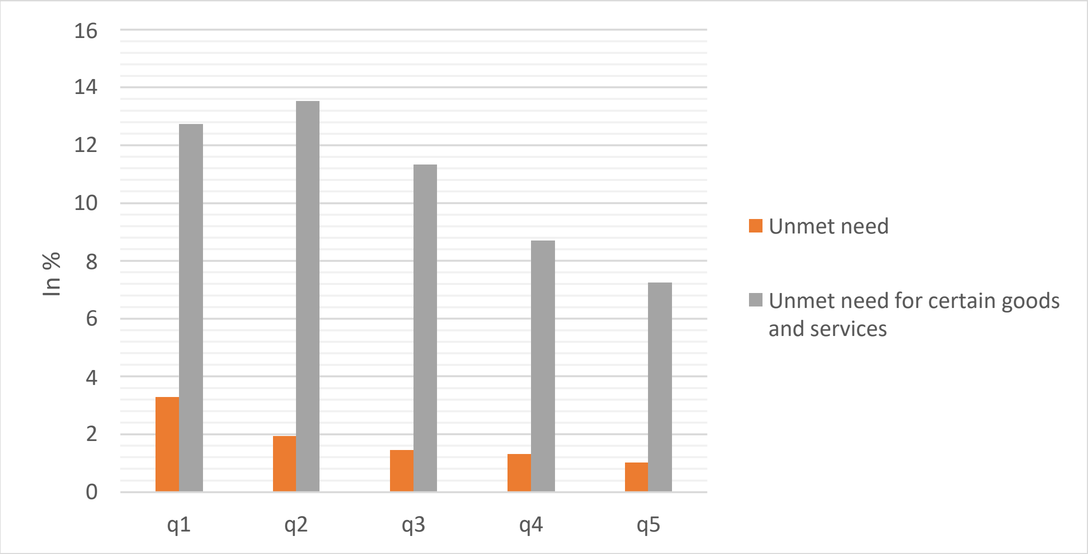

Figure S6. Percentage share of households with unmet need (defined as households who experience unmet need and incur zero healthcare expenditure) and unmet need for medicines and certain services, per income quintile, pooled RLMS data, 2010–2017.

Source/Notes: RLMS. The following values for the Pearson correlation coefficient are reported. In the case of unmet need and SES, Pearson chi2=131.27 (p=0.000), while in the case of unmet need for certain goods and services and SES, Pearson chi2=207.547 (p=0.000).
