# Supplementary material for: Financial Risk Protection and Unmet Healthcare Need in Russia
Source: Int J Health Policy Manag. 2021 Jul 14;11(9):1715–24. doi: 10.34172/ijhpm.2021.72 (PMC9808245; doi:10.34172/ijhpm.2021.72)
Supplement: Supplementary file 2 — Additional Results Based on the Longitudinal Sample. [file ijhpm-11-1715-s002.pdf]

Article title: Financial Risk Protection and Unmet Healthcare Need in Russia
Journal name: International Journal of Health Policy and Management (IJHPM)
Authors' information: Zlatko Nikoloski*, Jane Cheatley, Elias Mossialos
Department of Health Policy, London School of Economics and Political Science, London, UK.
(*Corresponding author: z.nikoloski@lse.ac.uk)

Supplementary file 2. Additional Results Based on the Longitudinal Sample

Table S5. Percentage share of households with catastrophic health care expenditure (measured as a share of total household consumption), pooled RLMS data, 2010-2017, longitudinal

	10% threshold	25% threshold	30% threshold	40% threshold
entire sample	6.30	1.19	0.75	0.34
2010	6.71	1.46	1.09	0.62
2011	6.83	1.18	0.68	0.28
2012	7.64	1.55	1.06	0.50
2013	6.83	1.49	0.90	0.28
2014	6.99	1.21	0.68	0.34
2015	5.50	1.18	0.87	0.47
2016	4.60	0.87	0.40	0.12
2017	5.28	0.56	0.31	0.09

Source/Notes: RLMS

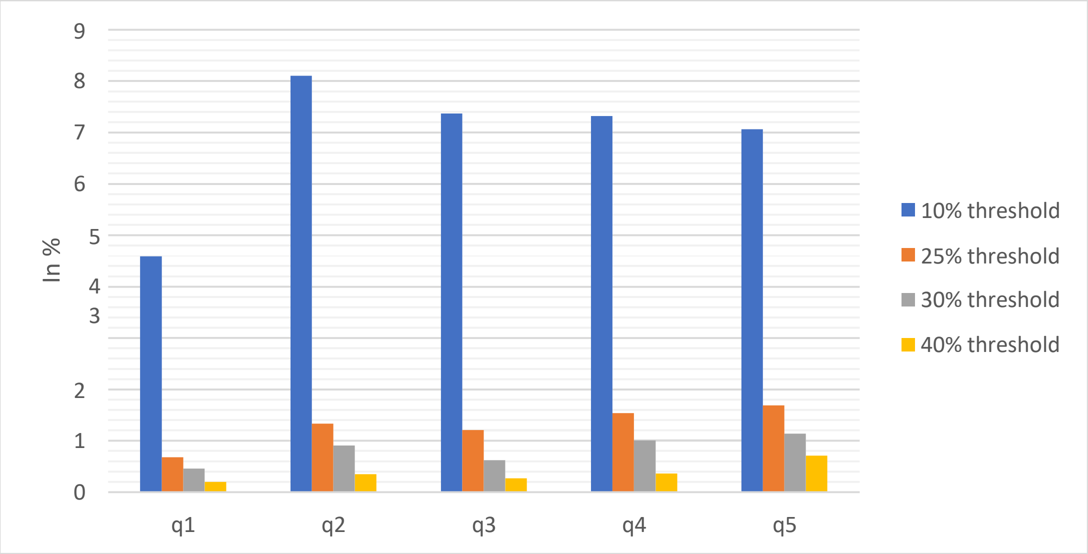

Figure S7. Percentage share of households with catastrophic health care expenditure (measured as a share of total household consumption) and assessed against the relevant threshold, per income quintile, pooled RLMS data, 2010–2017, longitudinal.

Source/Notes: RLMS. The following values for the Pearson chi2 were reported: for the link between SES and 10% - Pearson chi2=43.87 (p=0.000), SES and 25% CHE threshold – Pearson chi2=17.576 (p=0.000), SES and 30% CHE threshold – Pearson chi2=14.268 (p=0.000), SES and 40% CHE thresholds – Pearson chi2=14.163 (p=0.000).

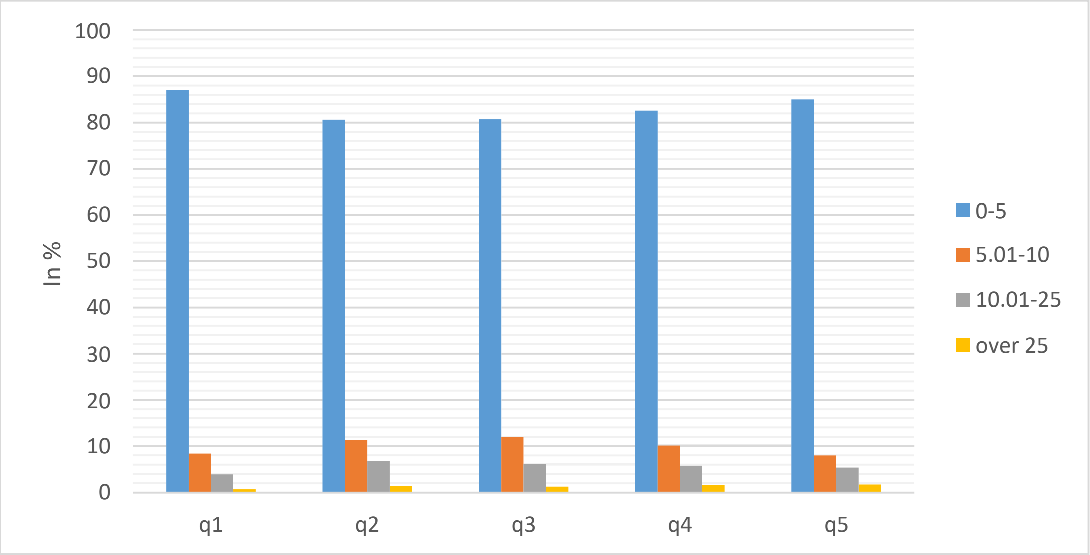

Figure S8. Distribution of households with respective expenditure on healthcare (as a share of total consumption), by income quintiles (in %), pooled RLMS data 2010–2017, longitudinal.

Source/Notes: RLMS. The following value for the Pearson coefficient are reported – Pearson chi2=107.112 (p=0.000).

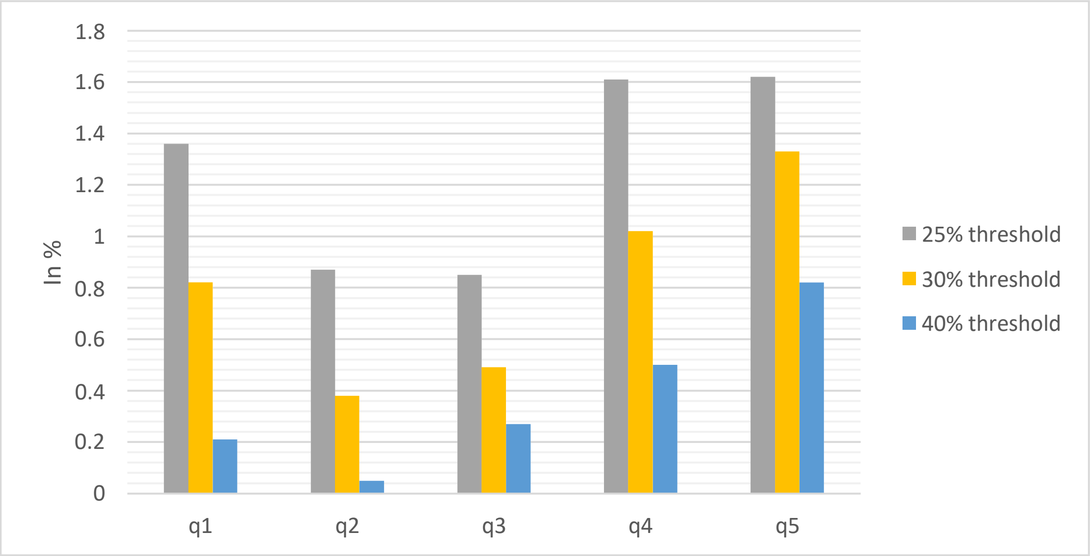

Figure S9. Percentage share of households with catastrophic health care expenditure (measured as a share of total household expenditure) and assessed against the relevant threshold, per consumption quintile, pooled RLMS data 2010–2017, longitudinal.

Source/Notes: RLMS. The following values for the Pearson coefficient are reported: 25% threshold – Pearson chi2=17.425 (p=0.002), 30% - Pearson chi2=28.058 (p=0.000), 40% - Pearson chi2=37.419 (p=0.000)

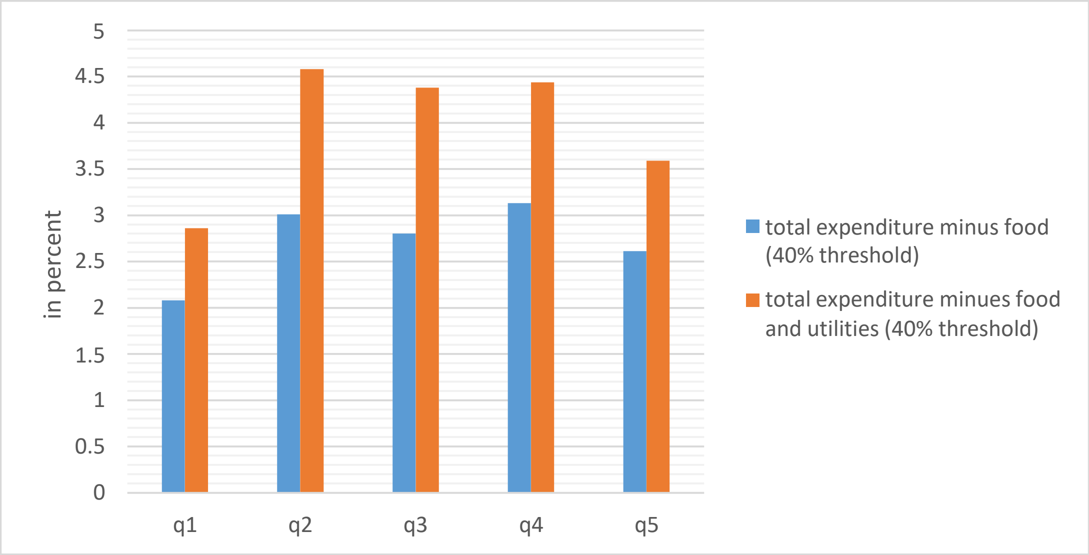

Figure S10. Percentage share of households with catastrophic health care expenditure (measured as a share of healthcare expenditure in total expenditure minus food and total expenditure minus food, rent and utilities), per income quintile, pooled RLMS data 2010–2017, longitudinal.

Source/Notes: RLMS. The following values for the Pearson coefficient were obtained. When using 40% threshold of total expenditure less food, Pearson chi2=9.86 (p=0.042), while when using 40% threshold of total expenditure less food and utilities, Pearson chi2=21.445 (p=0.000)

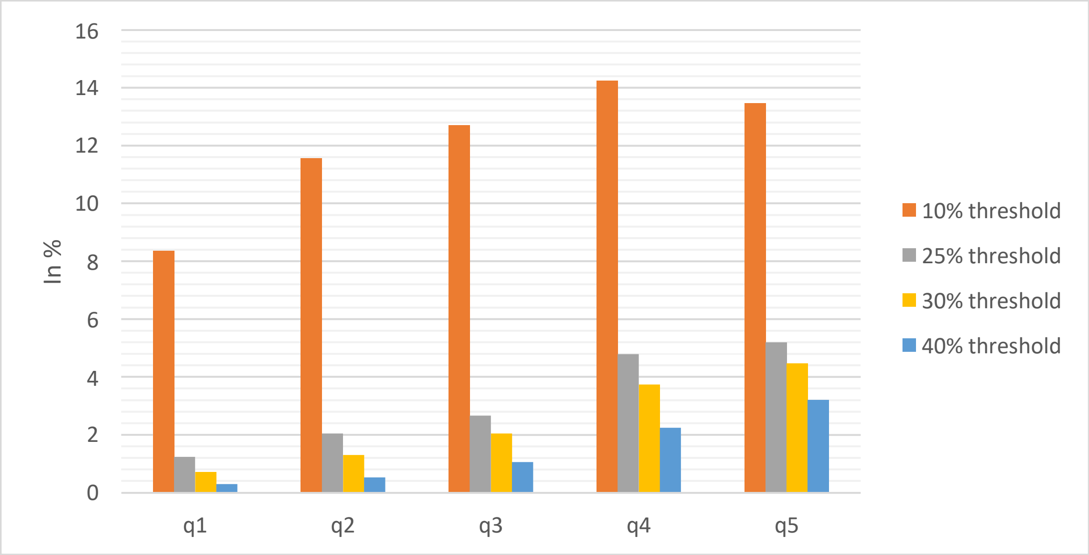

Figure S11. Percentage share of households with catastrophic health care expenditure (as a share of income), per consumption quintile, pooled RLMS data 2010–2017, longitudinal.

Source/Notes: RLMS. The following values for the Pearson coefficient were obtained on the link between SES and CHE. When using the 10% threshold, Pearson chi2=72.892 (p=0.000), when using 25%, Pearson chi2=144.098 (p=0.000), when using 30% threshold, Pearson chi2=157.38 (p=0.000) and when using the 40% threshold, Pearson chi2=153.93 (p=0.000)

Table S6. Overshoot and mean positive overshoot of the CHE (measured as a share of total household consumption) per income quintile, (in %), pooled RLMS, 2010-2017, longitudinal

	10% threshold	25% threshold	30% threshold	40% threshold
Overshoot	0.6	0.14	0.09	0.04
Mean positive overshoot	9.1	11.77	12.33	11.89
Overshoot by year				
	10% threshold	25% threshold	30% threshold	40% threshold
Overshoot - 2010	0.7	0.24	0.18	0.09
Mean positive overshoot - 2010	10.49	16.68	16.71	15.91
Overshoot - 2011	0.6	0.12	0.08	0.03
Mean positive overshoot - 2011	8.82	10.89	12.28	12.92
Overshoot - 2012	0.75	0.21	0.15	0.07
Mean positive overshoot - 2012	9.9	14.02	14.31	14.4
Overshoot - 2013	0.63	0.14	0.08	0.02
Mean positive overshoot - 2013	9.26	9.44	9.1	8.8
Overshoot - 2014	0.61	0.13	0.09	0.04
Mean positive overshoot - 2014	8.74	11.19	13.41	12.01
Overshoot - 2015	0.53	0.14	0.09	0.03
Mean positive overshoot - 2015	9.78	12.42	10.9	7.73
Overshoot - 2016	0.36	0.06	0.03	0.006
Mean positive overshoot - 2016	7.96	6.86	7.24	5.22
Overshoot - 2017	0.33	0.04	0.02	0.006

Mean positive overshoot - 2017	6.32	8.3	8.03	7.04

Source/Notes: RLMS

Table S7. Overshoot and mean positive overshoot of the CHE (measured as a share of total household consumption) per income quintile, (in %), pooled RLMS, 2010-2017, longitudinal

	10% threshold	25% threshold	30% threshold	40% threshold
Overshoot q1	0.37	0.07	0.04	0.02
Mean positive overshoot – q1	7.92	11.53	10.96	9.65
Overshoot - q2	0.7	0.1	0.1	0.04
Mean positive overshoot – q2	8.29	12.53	12.38	13.65
Overshoot - q3	0.6	0.1	0.08	0.04
Mean positive overshoot – q3	8.28	10.64	13.39	16.46
Overshoot - q4	0.7	0.2	0.1	0.05
Mean positive overshoot – q4	9.94	11.95	11.64	14.18
Overshoot - q5	0.7	0.2	0.16	0.07
Mean positive overshoot – q5	11.07	13.92	14.36	10.08

Source/Notes: RLMS. The following values for the Pearson chi2 were reported: for the link between SES and 10% overshoot- Pearson chi2=41.0 (p=0.000), SES and 25% overshoot – Pearson chi2=14.75 (p=0.005), SES and 30% overshoot – Pearson chi2=11.62 (p=0.02), SES and 40% overshoot – Pearson chi2=11.45 (p=0.02).

Table S8. Impoverishing effects of OOP (poverty headcount, poverty gap and normalized poverty gap), (in %), pooled RLMS, 2010-2017, longitudinal

Poverty headcount ratio gross of healthcare payments				Poverty headcount ratio net of healthcare payments				difference		
	1.9 USD per day, constant 2011, PPP	3.2 per day, constant 2011, PPP	5.5 USD per day, constant 2011, PPP		1.9 USD per day, constant 2011, PPP	3.2 per day, constant 2011, PPP	5.5 USD per day, constant 2011, PPP	1.9 USD per day, constant 2011, PPP	3.2 per day, constant 2011, PPP	5.5 USD per day, constant 2011, PPP
Poverty headcount	0.2	0.4	1.2	Poverty headcount	0.2	0.5	1.4	Poverty headcount	0.0	0.1	0.2
Poverty gap	0.9	1.9	3.6	Poverty gap	0.9	2.0	3.6	Poverty gap	0.0	0.0	0.1
Normalized poverty gap	0.5	0.6	0.6	Normalized poverty gap	0.5	0.6	0.7	Normalized poverty gap	0.0	0.0	0.0

Source/Notes: RLMS

Table S9. Impoverishing effects of OOP (poverty headcount, poverty gap and normalized poverty gap), (in %), pooled RLMS, 2010-2017, longitudinal

		1.9 USD per day, constant 2011, PPP	3.2 per day, constant 2011, PPP	5.5 USD per day, constant 2011, PPP			1.9 USD per day, constant 2011, PPP	3.2 per day, constant 2011, PPP	5.5 USD per day, constant 2011, PPP			1.9 USD per day, constant 2011, PPP	3.2 per day, constant 2011, PPP	5.5 USD per day, constant 2011, PPP
2010	Poverty headcount	0.3	0.5	1.1	2010	Poverty headcount	0.3	0.5	1.2	2010	Poverty headcount	0.0	0.0	0.1
	Poverty gap	1.1	1.9	3.3		Poverty gap	1.1	1.9	3.4		Poverty gap	0.0	0.0	0.1
	Normalized poverty gap	0.6	0.6	0.6		Normalized poverty gap	0.6	0.6	0.6		Normalized poverty gap	0.0	0.0	0.0
	Poverty headcount	0.1	0.4	1.0		Poverty headcount	0.1	0.4	1.1		Poverty headcount	0.0	0.0	0.1

	Poverty gap	0.8	2.1	3.5			Poverty gap	0.8	2.1	3.5			Poverty gap	0.0	0.0	0.0
2011	Normalized poverty gap	0.4	0.6	0.6		2011	Normalized poverty gap	0.4	0.6	0.6		2011	Normalized poverty gap	0.0	0.0	0.0
	Poverty headcount	0.2	0.5	1.1			Poverty headcount	0.2	0.5	1.2			Poverty headcount	0.0	0.0	0.2
	Poverty gap	0.9	2.1	3.4			Poverty gap	0.9	2.1	3.5			Poverty gap	0.0	0.0	0.1
2012	Normalized poverty gap	0.5	0.7	0.6		2012	Normalized poverty gap	0.5	0.7	0.6		2012	Normalized poverty gap	0.0	0.0	0.0
	Poverty headcount	0.2	0.4	0.8			Poverty headcount	0.2	0.4	1.0			Poverty headcount	0.0	0.1	0.2
	Poverty gap	0.8	1.9	3.1			Poverty gap	0.8	1.9	3.2			Poverty gap	0.0	0.0	0.2
2013	Normalized poverty gap	0.4	0.6	0.6		2013	Normalized poverty gap	0.4	0.6	0.6		2013	Normalized poverty gap	0.0	0.0	0.0
	Poverty headcount	0.2	0.2	0.6			Poverty headcount	0.2	0.2	0.9			Poverty headcount	0.0	0.0	0.2
	Poverty gap	0.8	1.2	3.4			Poverty gap	1.0	1.2	3.7			Poverty gap	0.1	0.0	0.2
2014	Normalized poverty gap	0.4	0.4	0.6		2014	Normalized poverty gap	0.5	0.4	0.7		2014	Normalized poverty gap	0.1	0.0	0.0
	Poverty headcount	0.2	0.4	1.6			Poverty headcount	0.2	0.5	1.9			Poverty headcount	0.1	0.1	0.3

	Poverty gap	0.6	1.7	3.6			Poverty gap	0.7	1.8	3.8			Poverty gap	0.2	0.0	0.2
2015	Normalized poverty gap	0.3	0.5	0.7		2015	Normalized poverty gap	0.4	0.5	0.7		2015	Normalized poverty gap	0.1	0.0	0.0
	Poverty headcount	0.2	0.5	2.0			Poverty headcount	0.2	0.7	2.4			Poverty headcount	0.0	0.2	0.4
	Poverty gap	0.9	2.0	3.8			Poverty gap	0.9	2.1	3.8			Poverty gap	0.0	0.1	0.0
2016	Normalized	0.5	0.6	0.7		2016	Normalized	0.5	0.6	0.7		2016	Normalized	0.0	0.0	0.0
	poverty gap						poverty gap						poverty gap			
	Poverty headcount	0.1	0.5	1.6			Poverty headcount	0.2	0.6	2.0			Poverty headcount	0.0	0.1	0.3
	Poverty gap	0.6	2.1	3.7			Poverty gap	0.8	2.1	3.8			Poverty gap	0.2	0.0	0.1
2017	Normalized poverty gap	0.3	0.6	0.7		2017	Normalized poverty gap	0.4	0.7	0.7		2017	Normalized poverty gap	0.1	0.0	0.0

Source/Notes: RLMS

Table S9. Percentage share of households with unmet need (as reported in the survey), by type of healthcare service, pooled RLMS data, 2010-2017, longitudinal

	Unmet dental care	Unmet pharmaceutical care	Unmet inpatient care	Unmet outpatient care
entire sample	9.16	6.79	3.75	2.99
2010	9.58	5.86	3.43	2.86
2011	6.77	5.48	3.15	2.22
2012				
2013	12.2	10.92	5.83	4.10
2014	8.88	5.84	3.04	2.56
2015	8.66	6.40	3.61	2.81
2016	9.82	7.12	3.35	2.97
2017	8.26	5.97	3.88	3.37

Source/Notes: RLMS. Note: the unmet need variables were not assessed in 2012.

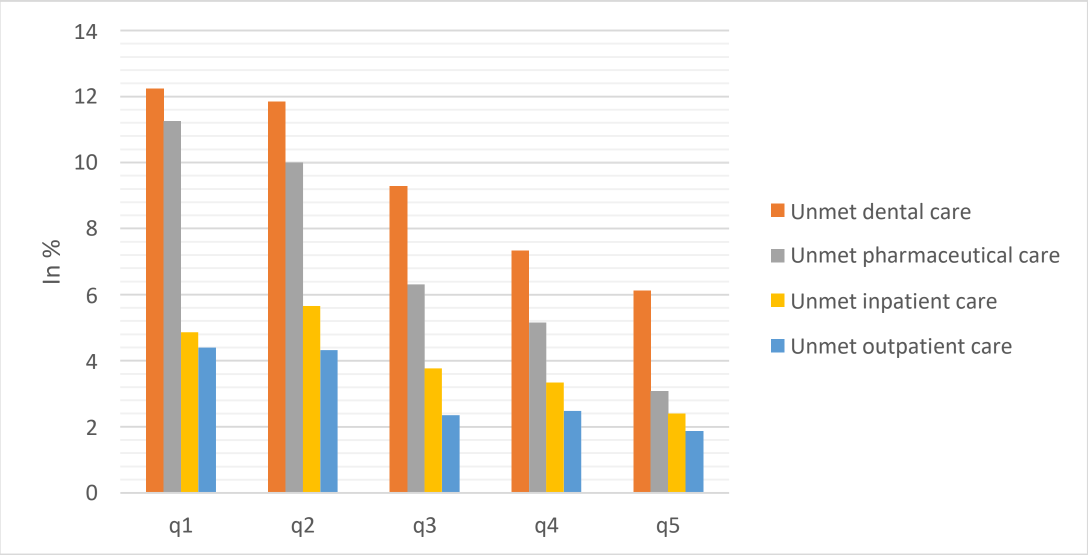

Figure S12. Percentage share of households with unmet need (as reported by the survey respondents), by income quintile and type of unmet need, pooled RLMS data 2010–2017, longitudinal.

Source/Notes: RLMS. The following values for the Pearson chi2 are reported. In case of unmet dental care and SES, Pearson chi2=78.28 (p=0.000), unmet need for medicines and SES, Pearson chi2=194.099 (p=0.000); unmet need for inpatient care and SES, Pearson chi2=36.33 (p=0.000); unmet need for outpatient care and SES, Pearson chi2=48.4

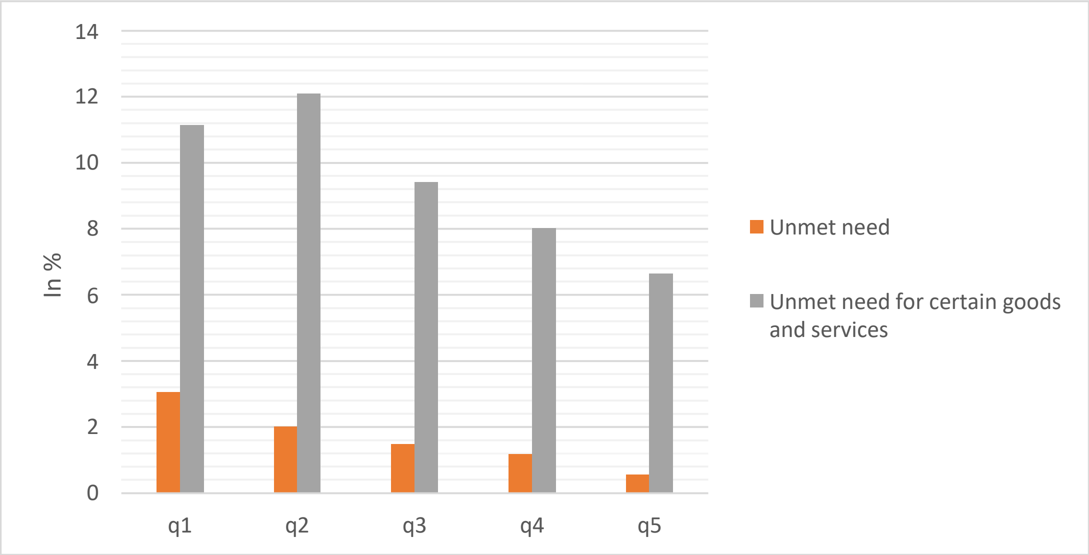

Figure S13. Percentage share of households with unmet need (defined as households who experience unmet need and incur zero healthcare expenditure) and unmet need for medicines and certain services, per income quintile, pooled RLMS data, 2010–2017, longitudinal.

Source/Notes: RLMS. The following values for the Pearson correlation coefficient are reported. In the case of unmet need and SES, Pearson chi2=66.85 (p=0.000), while in the case of unmet need for certain goods and services and SES, Pearson chi2=71.53 (p=0.000).07 (p=0.000).
